# Insufficient Post-operative Energy Intake Is Associated With Failure of Enhanced Recovery Programs After Laparoscopic Colorectal Cancer Surgery: A Prospective Cohort Study

**DOI:** 10.3389/fnut.2021.768067

**Published:** 2021-12-21

**Authors:** Shuang Liu, Sheng Zhang, Zike Li, Meng Li, Yujie Zhang, Min He, Chengcheng Jin, Chun Gao, Jianping Gong

**Affiliations:** ^1^Department of Gastrointestinal Surgery, Tongji Medical College, Tongji Hospital, Huazhong University of Science and Technology, Wuhan, China; ^2^Department of Pharmacy, Tongji Hospital, Tongji Medical College, Huazhong University of Science and Technology, Wuhan, China

**Keywords:** enhanced recovery after surgery, failure, post-operative activity, energy intake, laparoscopic colorectal surgery

## Abstract

**Background:** Although enhanced recovery after surgery (ERAS) has been proven to be beneficial after laparoscopic colorectal surgery, some of the patients may fail to complete the ERAS program during hospitalization. This prospective study aims to evaluate the risk factors associated with ERAS failure after laparoscopic colorectal cancer surgery.

**Methods:** This is a prospective study from a single tertiary referral hospital. Patients diagnosed with colorectal cancer who met the inclusion criteria were included in this study. Demographic and clinicopathological characteristics were collected. Post-operative activity time and 6-min walking distance (6MWD) were measured. Patients were divided into ERAS failure group and ERAS success according to decreased post-operative activity and 6MWD. Factors associated with ERAS failure were investigated by univariate and multivariate analysis.

**Results:** A total of 91 patients with colorectal cancer were included. The incidence of ERAS failure is 28.6% among all patients. Patients in ERAS failure group experienced higher rate of post-operative ileus and prolonged hospital stay (*p* < 0.001). Multivariate analysis revealed that older age (*p* = 0.006), body mass index ≥25.5 kg/m^2^ (*p* = 0.037), smoking (*p* = 0.002), operative time (*p* = 0.048), and post-operative energy intake <18.5 kcal/kg•d (*p* = 0.045) were independent risk factors of ERAS failure after laparoscopic colorectal surgery.

**Conclusions:** Our findings indicated that a proportion of patients may fail the ERAS program after laparoscopic colorectal surgery. We for the first time showed that post-operative energy intake was an independent risk factor for ERAS failure. This may provide evidence for further investigation on precise measurement of nutritional status and selected high-risk patients for enhanced nutrition support.

## Introduction

The principles of enhanced recovery after surgery (ERAS), first proposed in 1997 by Kehlet and Wilmore (1) were well-established in perioperative management. Aims to improve clinical outcomes and accelerate post-operative recovery after surgery, this multimodal approach was proved to be effective in numerous clinical trials (2–4). ERAS program contains various managements including pre-operative patient education, optimized anesthesia, pre-operative and post-operative medicine with pain management, post-operative antiemetic, fluid restriction, no surgical drains, no standard post-operative nasogastric tubes, post-operative nutritional care, early oral intake, and early mobilization (5, 6).

Studies have shown that the successful application of ERAS has a significant impact on post-operative outcomes and reduces hospital stay after laparoscopic colorectal surgery (7, 8). However, in clinical practice, compliance with core components in ERAS protocols is challenging for both clinical professionals and patients. Poor compliance results in failure of ERAS management and may lead to delayed discharge after surgery (9, 10). The reasons for failure to comply with elements in the ERAS program were investigated and reported from several studies. These factors included demographic characteristics such as older age, male gender, and previous abdominal operation history, intraoperative factors such as more blood loss and longer operation time, and post-operative factors such as surgical complications and reoperation (11). It is important to identify and modulate risk factors to avoid deviation or failure of the ERAS program.

Failure of ERAS may be evaluated as incompliance of ERAS components or prolonged length of hospital stay. Studies showed that early mobilization was an independent determinant of early recovery and significantly associated with a successful outcome of ERAS after laparoscopic colorectal surgery (12, 13). Six-min walking distance (6MWD) is a simple and reliable test to measure post-operative function capacity. 6MWD was a predictive factor of post-operative complications and length of stay (14, 15). Measured by 6MWD, decreased post-operative activity can be an important indicator for the failure of ERAS.

Therefore, we conducted a prospective study to investigate whether post-operative functional capacity can be a predictive factor for the failure of the ERAS program. Meanwhile, we also analyzed the risk factors for decreased post-operative functional capacity in patients who underwent laparoscopic colorectal surgery.

## Methods

### Study Design and Participants

We conducted a prospective cohort study of 91 consecutive patients with colorectal cancer admitted to our department from February 2018 to December 2018. The work has been reported in line with the strengthening the reporting of cohort studies in surgery (STROCSS) criteria (16). Patients aged over 18 years who met the following inclusion criteria were recruited in this study: (1) patients underwent elective laparoscopic colorectal surgery; (2) histopathological confirmed diagnosis of colorectal cancer; (3) age <70 years old; (4) patients had no history of diabetes mellitus or paralysis that may affect post-operative recovery; and (5) patients who were willing to cooperate in the enhanced recovery programs. The exclusion criteria were: (1) patients with severe cardiovascular and cerebrovascular diseases; (2) patients with IV stage of disease or underwent palliative surgery; and (3) patients that were unwilling to cooperate in the enhanced recovery programs.

This study was approved by the institutional medical ethics committee (TJ-20170803), with all aspects in this study complying with the Helsinki Declaration. Informed consent was signed by all participants. The study was conducted and presented in compliance with the strengthening of the reporting of cohort studies in surgery (STROBE) guideline (Supplementary Table 1) (16).

### Data Collection

The following data were collected for analysis: (1) demographic characteristics (age, sex, body mass index (BMI), medical history, and tumor localization); (2) laboratory characteristics (albumin, pre-albumin, hemoglobin, transferrin, retinol-binding protein, etc.); (3) intraoperative data (operative type, duration of surgery, blood loss, and stoma); and (4) post-operative data (energy intake, tumor stage, mortality, surgical complications, and length of stay).

### Monitoring of Post-operative Activity Time and 6MWD

In all patients, post-operative activity time was monitored from post-operative day 1 to discharge and 6MWD was monitored from pre-operative day 1 to discharge. Post-operative activity time was measured as follows: each patient was given a pedometer to record the daily time of getting out of bed after surgery. 6MWD was measured according to guidelines (17). The goal of the test is to walk as far as possible for 6 min. Patients will walk back and forth in a hallway, they are permitted to slow down, to stop, and to rest as necessary, but resume walking as soon as able. The 6MWD was monitored at the same time every day. We define the failure of ERAS as a decline of more than 10% of post-operative activity time or 6MWD from the previous day. Patients were divided into the ERAS success group and the ERAS failure group according to the definition.

### Nutritional Support Program

Nutrition support is an essential component of ERP. And it has been accepted that early oral nutrition in the post-operative period could promote early recovery (18). Therefore, our protocol aimed to initiate enteral nutrition or oral nutrition as early as possible, and supplemental parenteral nutrition was carefully applied considering the progress of the patient. The nutritional intervention is outlined in Supplementary Table 2. More specifically, the nutritional support program was divided into four phases. Phase I implemented before the operation, patients received 400 ml of oral carbohydrates (complex CHO-maltodextrin, 12.5%, 285 mOsm/kg) in the evening before surgery and 2–4 h before induction of anesthesia. Phase II commenced on the first day after surgery and ended after the first exhaust. During this phase, patients were encouraged to take liquid food, and oral nutritional supplements (ONS) (100 ml = 200 kcal + 4 g protein) were also provided to patients, which were encouraged to drink in small, frequent quantities between meals. Phase III referred to 2 days after the first exhaust. Patients predominantly took an oral semisolid diet and ONS were gradually increased. Phase IV began at the end of phase III and ended after discharge. Patients in this phase resumed regular oral diets. If any patient showed an inability to tolerate an oral diet, the intake was withheld instantly. The criteria for intolerance were defined as any of the following: abdominal distension, moderate to severe pain (Likert score > 3/5), vomiting, or diarrhea. The oral diet would be reintroduced once the symptoms had completely receded. PN was provided according to guidelines from the European Society for Clinical Nutrition and Metabolism (ESPEN), which recommend the application of post-operative PN for patients who cannot meet their energy needs orally/enterally within 5–7 days (19). The recommended intake of protein was 1.5 g/kg ideal body weight. The dietary intake was recorded on a daily food chart by the research staff.

### Statistical Analysis

We presented the continuous variables as mean (SD)/medians [interquartile range (IQR)] and analyzed them with student unpaired *t*-test or Mann–Whitney *U*-test. Receiver operating characteristic (ROC) analysis was applied to acquire the area under the curve. The ROC-derived optimal cutoff was determined at the maximal Youden index. We reported categorical variables as whole numbers and percentages and analyzed them with the chi-squared test or the Fisher's exact test. We applied the univariate logistic regression to evaluate potential risk factors for ERAS failure. Only factors with a *P* < 0.1 in univariate analysis were included in the final multivariate analysis model. Multivariate logistic regression was employed to identify independent risk factors for the failure of ERAS. All *p*-values were reported as two-sided with a significance level of 0.05. Analyses were conducted using SPSS version 24.0 (IBM, Armonk, NY, USA).

## Results

### Demographics and Post-operative Functional Capacity of Patients With Colorectal Cancer

**Figure 1:**
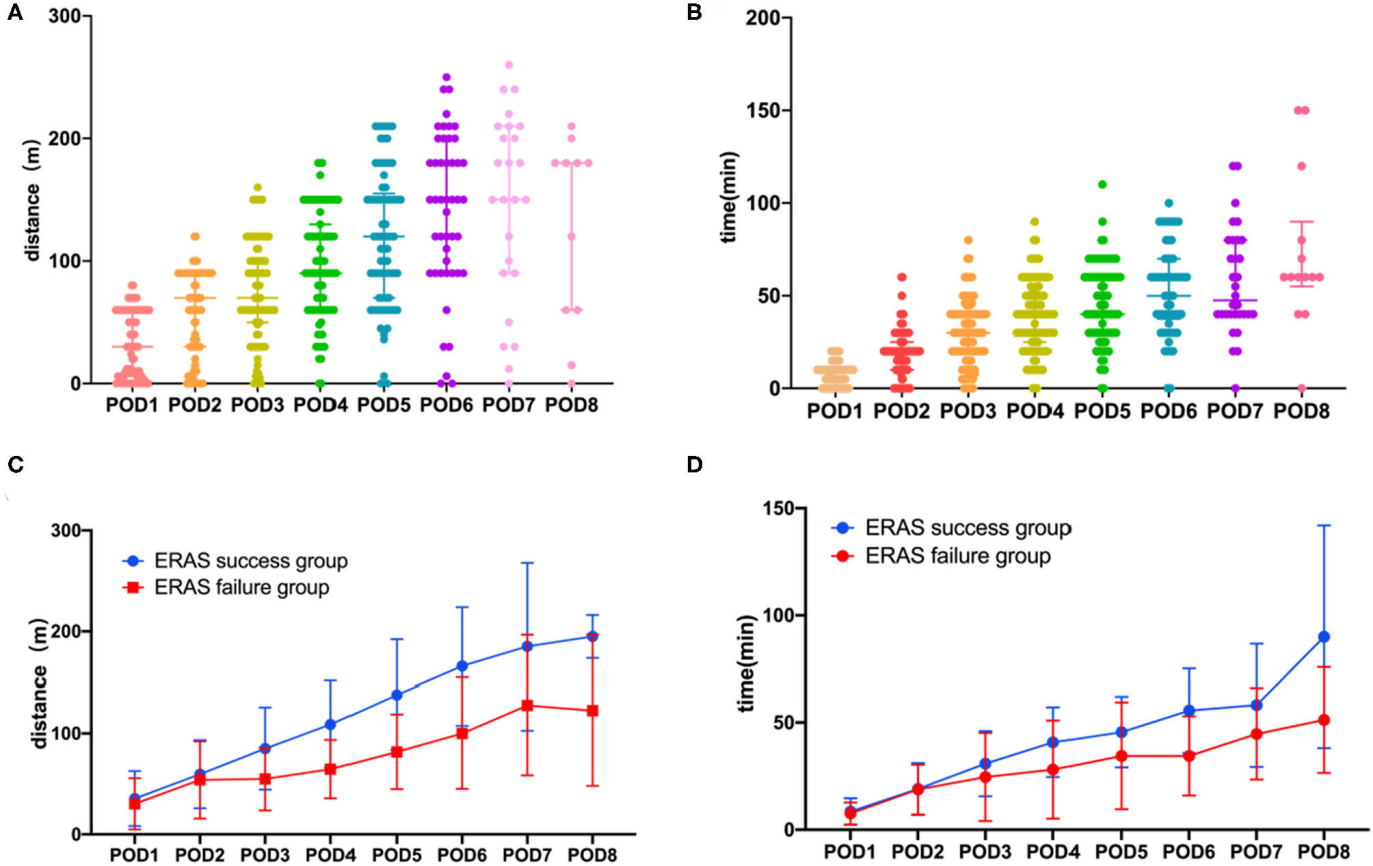
Post-operative functional capacity of patients. **(A)** Six minutes walking distance in all the patents. **(B)** Post-operative activity time in all the patients. **(C)** Six minutes walking distance in ERAS success group and ERAS failure group. **(D)** Post-operative activity time in ERAS success group and ERAS failure group. Median and IQR are shown for all scatter plots and Mann–Whitney tests were performed to compare differences between groups.

A total of 91 consecutive patients who underwent laparoscopic colorectal surgery were recruited, comprising 45 men (49.5%) and 46 women (50.5%). The median age was 54 years old (IQR: 48–60). Figures 1A,B demonstrated the characteristics of post-operative activity time and 6MWD of included patients. According to the definition, ERAS failure occurred in 26 (28.6%) patients. As shown in Table 1, patients in the ERAS failure group were more likely to have a history of smoking (*p* = 0.011). There were no significant differences between the ERAS success group and failure group in terms of gender, BMI, history of abdominal history, comorbidities, and type of cancer. Other information was summarized in Table 1. Pooled values of post-operative activity time and 6MWD on post-operative days 1–8 were quantified in Table 1 and illustrated in Figures 1C,D. The number and percentage of patients define as ERAS failure on each post-operative day were summarized in Supplementary Table 3. According to the definition, we observed a significant difference in both post-operative activity time and 6MWD between ERAS success and failure groups. An interesting notion is that the differences between groups appeared between post-operative days 4–6. Thereafter, the failure group achieves a similar activity time on POD8. As for 6MWD, although the differences between groups appeared between post-operative days 3–7, the increase in 6MWD is parallel in both groups.

**Table 1:** The demographic and laboratory characteristics of patients with colorectal cancer.

**Variables**	**All patients (*N* = 91)**	**ERAS success group (*n* = 65)**	**ERAS failure group (*n* = 26)**	***p* value**
Age, median (IQR), years	54 (48, 60)	53 (47, 59)	53 (47, 59)	0.174
Gender, male, *n* (%)	45 (49.5%)	32 (49.2%)	13 (50.0%)	0.947
Smoking, yes, *n* (%)	11 (12.1%)	4 (6.2%)	7 (26.9%)	**0.011**
BMI, median (IQR), kg/m^2^	22.1 (20.2, 24.9)	22.0 (20.2, 24.3)	22.2 (20.1, 25.7)	0.704
**History of abdominal surgery**				0.737
No	79 (86.8%)	57 (87.7%)	22 (84.6%)	
Yes	12 (13.2%)	8 (12.3%)	4 (15.4%)	
**Comorbidities**				0.077
No	63 (69.2%)	49 (75.4%)	14 (53.8%)	
Yes	28 (30.8%)	16 (24.6%)	12 (46.2%)	
**Type of cancer**				0.062
Colon	47 (51.6%)	38 (58.5%)	9 (34.6%)	
Rectal	44 (48.4%)	27 (41.5%)	17 (65.4%)	
**Laboratory characteristics**				
Albumin, g/l	42.0 (38.9–44.0)	42.0 (39.7–43.9)	42.4 (38.7–44.4)	0.978
Pre-Albumin, mg/l	223.0 (188.0–260.0)	227.0 (190.0–261.0)	211.0 (174.3–259.5)	0.490
Hemoglobin, g/l	124.0 (112.0–138.0)	124.0 (113.5–134.0)	119.0 (103.8–141.3)	0.548
Transferrin, g/l	2.4 (2.0–2.6)	2.3 (1.9–2.7)	2.4 (2.3–2.7)	0.363
Retinol-Binding protein, mg/l	36.2 (27.9–41.4)	34.5 (24.5–41.4)	39.4 (31.0–42.8)	0.402
**6MWD, median (IQR), m**				
POD1	30 (6, 60)	40 (8, 60)	30 (5.25, 60)	0.427
POD2	70 (30, 90)	70 (30, 90)	60 (10.25, 90)	0.48
POD3	70 (50, 100)	90 (60, 120)	60 (30, 62.5)	**0.001**
POD4	90 (60, 130)	120 (80, 150)	65 (37.5, 90)	**0.0001**
POD5	120 (70, 155)	150 (90, 180)	90 (60, 112.5)	**0.0001**
POD6	150 (90, 200)	180 (115, 210)	120 (52.5, 150)	**0.001**
POD7	150 (90, 210)	215 (135, 240)	150 (70, 190)	**0.035**
POD8	180 (60, 180)	N/A	150 (60, 180)	0.601
**Activity time, median (IQR), min**				
POD1	10 (5, 10)	10 (5, 10)	10 (5, 10)	0.668
POD2	20 (10, 30)	20 (10, 25)	20 (9.5, 30)	0.66
POD3	30 (20, 40)	30 (20, 40)	20 (8.75 40)	0.068
POD4	40 (20, 50)	40 (30, 50)	20 (10, 33.75)	**0.001**
POD5	40 (30, 60)	40 (30, 60)	30 (20, 40)	**0.005**
POD6	45 (30, 60)	50 (40, 65)	30 (23.75, 48.75)	**0.001**
POD7	40 (40, 65)	50 (40, 75)	40 (30, 65)	0.358
POD8	60 (40, 70)	N/A	60 (40, 67.5)	0.283

*For p-value: Boldface type indicates significant difference*.

*BMI, body-mass index; IQR, interquartile range; POD, post-operative day*.

### Perioperative Characteristics Between ERAS Success and Failure Patients

Perioperative characteristics were demonstrated in Table 2. There were no significant differences in the type of operative, operative time, blood loss, rate of stoma, and disease stage. Besides, we monitor the average post-operative energy intake, including supplemental parenteral nutrition and oral nutrition of included patients, and found that patients with ERAS failure had a significantly lower average energy intake (18.3, IQR 15.5–20.7 kcal/kg/day vs. 18.8, IQR 17.1–22.9 kcal/kg/day, *p* = 0.04).

**Table 2:** Operative and post-operative characteristics of patients with colorectal cancer.

**Variables**	**Median (IQR)**			***p*-value**
	**All patients (*N* = 91)**	**ERAS success group (*n* = 65)**	**ERAS failure group (*n* = 26)**	
**Operative**				
**Type of operation**				
Laparoscopic right hemicolectomy	28 (30.8%)	16 (24.6%)	14 (53.8%)	
Laparoscopic left hemicolectomy	6 (6.6%)	6 (9.2%)	0 (0%)	0.059
Laparoscopic rectal cancer resection	57 (62.6%)	43 (66.2%)	12 (46.2%)	
**Operative time, min**				
≤ 215 min	39 (42.9%)	34 (52.3%)	5 (19.2%)	**0.005**
>215 min	52 (57.1%)	31 (47.7%)	21 (80.8%)	
Blood loss, ml	22 (18, 23.5)	21 (17.8–23.3)	22 (20.8–23.5)	0.301
**Stoma**				
No	68 (74.7%)	48 (73.8%)	20 (76.9%)	0.760
Yes	23 (25.3%)	17 (26.2%)	6 (23.1%)	
**Post-Operative**				
Post-Operative average energy intake, kcal/kg/day	18.4 (16.9, 21.7)	18.8 (17.1, 22.9)	18.3 (15.5, 20.7)	**0.030**
**TNM stage**				
II	71 (78.0%)	52 (80.0%)	19 (73.1%)	0.471
III	20 (22.0%)	13 (20.0%)	7 (26.9%)	
Mortality	0 (0, 0)	0 (0, 0)	0 (0, 0)	1.000
**Overall complications**				
Fever	2 (2.2%)	1 (1.5%)	1 (3.8%)	0.556
Pulmonary infection	4 (4.4%)	2 (3.1%)	2 (7.7%)	0.643
Post-Operative ileus	29 (28.6%)	15 (23.1%)	14 (53.8%)	**0.004**
Wound infection	9 (9.9%)	6 (9.2%)	3 (11.5%)	0.885
Intra-Abdominal hemorrhage	5 (5.5%)	3 (4.6%)	2 (7.7%)	0.722
Post-Operative length of stay, median, day	6(5,7)	5(4,6)	7 (5,9)	**0.002**

*For p-value: Boldface type indicates significant difference*.

*TNM, tumor-node-metastasis*.

**Figure 2:**
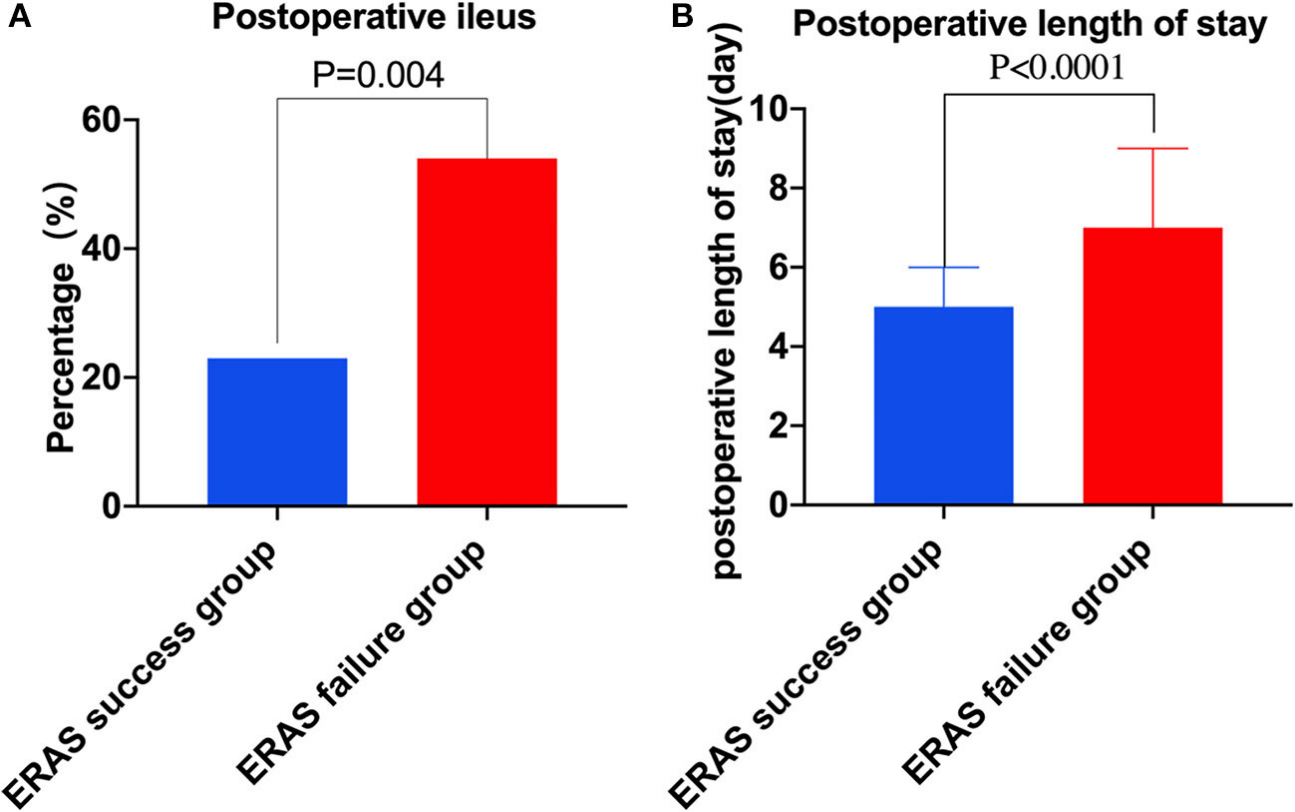
Complications and post-operative length of stay in two groups. **(A)** Post-operative ileus in two groups. **(B)** Post-operative length of stay in two groups.

In short-term post-operative outcomes, patients in the ERAS failure group experienced a higher rate of post-operative ileus (53.8 vs. 23.1%, *p* = 0.004) compared to the ERAS success group (Figure 2A). Moreover, patients in the ERAS failure group had a significantly longer length of post-operative hospital stay (7 days, IQR 5–9 vs. 5 days, IQR 4–6 days, *p* = 0.002; Table 2, Figure 2B).

### Risk Factors Associated With Decreased Post-operative Functional Capacity

Risk factors for ERAS failure identified from univariate analysis were shown in Table 3. Univariate analysis revealed that ERAS failure was associated with older age (≥55 vs. <55 yrs, hazard ratio (HR) = 3.23, *p* = 0.016), overweight (BMI ≥ 25.5 vs. BMI <25 kg/m^2^, HR = 2.77, *p* = 0.067), smoking (yes vs. no, HR = 5.62, *p* = 0.011), comorbidities (yes vs. no, HR = 2.63, *p* = 0.048), type of cancer (rectal vs. colon, HR = 2.66, *p* = 0.043), operative time (≤ 215 vs. >215 min, HR = 3.83, *p* = 0.016), operative type (right colon vs. left colon or rectal, HR = 2.63, *p* = 0.048), Post-operative ileus (yes vs. no, HR = 3.889, *p* = 0.006), post-operative energy intake (≥18.5 vs. <18.5 kcal/kg•d, HR = 0.16, *p* = 0.020). Multivariate analysis showed that older age (*p* = 0.006), BMI ≥ 25.5 kg/m^2^ (*p* = 0.037), smoking (*p* = 0.002), operative time (>215 min) (*p* = 0.048), and post-operative energy intake <18.5 kcal/kg• d (*p* = 0.045) were independent risk factors for ERAS failure (Table 3).

**Table 3:** Univariate and multivariate analysis for ERAS failure.

**Variable**	**Univariate HR (95% CI)**	***p*-value**	**Multivariate HR (95% CI)**	***p*-value**
**Age**				
<55 yrs	1 (Ref)	**0.016**	1 (Ref)	**0.006**
≥55 yrs	3.23 (1.25–8.36)		6.36 (1.68–24.01)	
**Gender**				
Male	1 (Ref)	0.947		
Female	0.97 (0.39–2.41)			
**Body mass index**				
<25.5 kg/m^2^	1 (Ref)	**0.067**	1 (Ref)	**0.037**
≥25.5 kg/m^2^	2.77 (0.93–8.23)		4.54 (1.09–18.84)	
**Smoking**				
No	1 (Ref)	**0.011**	1 (Ref)	**0.002**
Yes	5.62 (1.48–21.29)		17.62 (2.85–108.95)	
**Comorbidities**				
No	1 (Ref)	**0.048**		NS
Yes	2.63 (1.01–6.83)			
**Type of cancer**				
Colon	1 (Ref)	**0.043**		NS
Rectal	2.66 (1.03–6.85)			
**Albumin**				
≥35 g/l	1 (Ref)	0.665		
<35 g/l	1.64 (0.17–15.40)			
**Pre-albumin**				
≥200 mg/l	1 (Ref)	0.460		
<200 mg/l	0.66 (0.21–2.01)			
**Operative time**				
≤ 215 min	1 (Ref)	**0.016**	1 (Ref)	**0.048**
>215 min	3.83 (1.29–11.39)		4.19 (1.01–17.32)	
**Operative type**				
Left colon or rectal	1 (Ref)	**0.048**		NS
Right colon	2.63 (1.01–6.83)			
**Stoma**				
No	1 (Ref)	0.760		
Yes	0.85 (0.29–2.46)			
**TNM stage**				
II	1 (Ref)	0.473		
III	1.47 (0.51–4.25)			
**Post-operative ileus**				
No	1 (Ref)	**0.006**		NS
Yes	3.889 (1.485–10.187)			
**Post-operative average energy intake**				
<18.5 kcal/kg d	1 (Ref)	**0.020**	1 (Ref)	**0.045**
≥18.5 kcal/kg d	0.16 (0.04–0.75)		0.16 (0.03–0.95)	

*For p-value: Boldface type indicates significant difference*.

*HR, hazard ratio; TNM, tumor-node-metastasis, NS, not significant*.

## Discussion

In this study, we performed a prospective study to investigate the factors associated with the failure of the ERAS program after laparoscopic colorectal surgery. Among 91 included patients, we found the rate of ERAS failure is 28.6%. Furthermore, we for the first time showed that post-operative energy intake is an independent risk factor for failure of ERAS.

Enhanced recovery after surgery (ERAS) program achieved great success in reducing the post-operative length of stay, total complications, perioperative morbidity, and even improving 5-year survival in laparoscopic colorectal surgery (7–9, 12, 13). Despite these advances, studies showed that only about a half of patients could complete post-operative recovery courses as prescribed by ERAS (9–11). Similarly, according to post-operative activity time and 6MWD, 26 patients (28.6%) who underwent laparoscopic colorectal cancer surgery could not achieve the aim of ERAS and therefore were categorized as ERAS failure group. Prolonged bed rest and decreased post-operative activity were associated with post-operative pulmonary and thromboembolic complications (20, 21). In our study, patients in the ERAS failure group also had a higher rate of post-operative ileus and suffered from prolonged hospital stay compared to the ERAS success group.

Diverse evaluations of ERAS failure have been proposed in different studies. Unplanned readmission within 30 days after surgery, prolonged hospital stay, and withdrawal from the ERAS program due to post-operative complications were different definitions of ERAS failure in various studies (22, 23). We applied post-operative activity and 6MWD as the measurement of ERAS failure in our study. To the best of our knowledge, this study for the first time to report these novel measures to define the clinical deterioration after ERAS.

According to the current literature, miscellaneous risk factors were reported to be associated with ERAS failure. Deborah et al. found that pre-operative anxiety, chronic pain, and intraoperative conversion were independent risk factors for failure of ERAS in a patient who underwent laparoscopic colorectal resections (24). Renz et al. reported that patients that are older age, increasing BMI, blood loss (>500 ml), pre-operative albumin level were associated with failure of ERAS in a patient undergoing elective colorectal surgery (25). Zhang et al. found that advanced age (>80 years old), history of abdominal surgery, and gastrointestinal obstruction were the risk factors for ERAS failure in patients who underwent gastric and colorectal cancer surgery (23). In our study, multivariate analysis showed that older age (≥55 yrs), history of smoking, higher BMI (≥25.5 kg/m^2^), longer operative time (>215 min) were independent risk factors associated with ERAS failure.

Cancer-related malnutrition is common in patients with malignant diseases (26). Malnutrition not only had a negative impact on treatment but also could be an important risk factor for post-operative morbidity and mortality (27). Suffering from eating disorders and disturbances, patients with gastrointestinal cancer usually had a high probability of malnutrition (28). A prospective observational study showed that within an ERAS program for colorectal cancer surgery, malnourished patients were at risk for increased post-operative morbidity, delayed recovery of gastrointestinal function, and prolonged length of hospital stay (29). Besides, studies have shown that despite the early post-operative oral nutritional support, patients who underwent major abdominal surgery still generally received underreporting energy and protein supply. Insufficient energy and protein intake were closely related to poor clinical outcomes (30, 31). A previous study from Yeung found that patients with ERAS consumed more protein and protein intakes were associated with reduced length of stay in elective colorectal surgery (32). In our present study, we found that inadequate energy intake was an independent risk factor associated with ERAS failure. Patients included did not have nutritional risk before surgery screened by NRS2002. Due to abdominal complications such as post-operative ileus, some of the patients could not tolerate the nutritional supplementation. This resulted in insufficient energy and protein intake and led to slow recovery and prolonged length of stay. To enhance the efficiency of nutritional support, several studies have proposed approaches to improve post-operative energy intake, such as standard use of fortified diets (e.g., high protein and high energy) (30). The efficiency of different nutritional interventions and their relationships to clinical outcomes need to be explored in future studies.

Since energy intake is closely correlated with post-operative recovery, accurate assessment of the nutritional status of patients and nutritional support would be crucial for the successful implementation of the ERAS program. Our previous study exhibited that the apoptosis/proliferation ratio of oral mucosal epithelial varied with the change of body nutritional status (33). This novel nutrition assessment is highly consistent with traditional serological measures and more sensitive in representing nutritional status. The application of the above measurement can precisely assess the nutritional status of patients and guide nutritional support.

The present study has several limitations that should be taken into consideration. First, the insufficient sample size might lead to deviation in results. Second, 6MWD and post-operative activity were novel measurements to evaluate the outcome of ERAS and the measurements were made before patients were discharged from the hospital. In addition, the definition of ERAS failure according to the decline of 6MWD or activity time was relatively subjective. The cut-off value of average post-operative energy intake in this study may not be applied in other studies. Third, some clinical data were not included in the analysis. Therefore, a prospective study with larger sample size and more variables included for analysis are needed to validate the finding of our study.

## Conclusions

During the laparoscopic era in colorectal surgery, failure of ERAS occurred in a certain proportion of patients, which may eventually lead to a prolonged length of stay. Our study for the first time indicated that insufficient energy intake was an independent risk factor of ERAS failure. Therefore, accurate assessment of nutritional status and nutritional support were critical for the successful implementation of the ERAS program.

## Data Availability Statement

The original contributions presented in the study are included in the article/Supplementary Material, further inquiries can be directed to the corresponding author/s.

## Ethics Statement

The studies involving human participants were reviewed and approved by Tongji Hospital Ethics Committee (TJ-20170803). The patients/participants provided their written informed consent to participate in this study.

## Author Contributions

CG and JG: conceptualization. SL, SZ, and CG: writing—original draft. SL and SZ: data curation, methodology, and software. SL, SZ, ML, and YZ: formal analysis and project administration. ZL, MH, and CJ: data collection. All authors participated in the study design, agreed on the final version, and meet the major criteria recommended by the ICMJE (http://www.icmje.org/).

## Funding

This research was supported by the National Science Funding of China (Grant No. 81272623) to CG.

## Conflict of Interest

The authors declare that the research was conducted in the absence of any commercial or financial relationships that could be construed as a potential conflict of interest.

## Publisher's Note

All claims expressed in this article are solely those of the authors and do not necessarily represent those of their affiliated organizations, or those of the publisher, the editors and the reviewers. Any product that may be evaluated in this article, or claim that may be made by its manufacturer, is not guaranteed or endorsed by the publisher.
