# Supplementary material for: Insufficient Post-operative Energy Intake Is Associated With Failure of Enhanced Recovery Programs After Laparoscopic Colorectal Cancer Surgery: A Prospective Cohort Study
Source: Front Nutr. 2021 Dec 21;8:768067. doi: 10.3389/fnut.2021.768067 (PMC8724790; doi:10.3389/fnut.2021.768067)
Supplement: Supplementary file 2 [file Table_2.docx]

Supplementary Table 2. Enhanced recovery after surgery protocol.

| Period | Items | Details |
| --- | --- | --- |
| Preoperative | Preoperative information and education | Detailed clinical information of every patient was recorded. And patients received dedicated preoperative education to cooperate with ERP and reduce anxiety. |
|  | Preoperative optimization | Preoperative optimization includes smoking cessation, avoiding alcohol abuse, and risk assessment. |
|  | Nutrition risk screening | The nutritional risk was determined using the Nutritional Risk Screening score (NRS 2002). |
|  | Preoperative nutrition | Patients at risk of malnutrition should receive oral nutritional supplementation (or additional parenteral nutrition) for at least 7–10 days before surgery. |
|  | Bowel preparation | Mechanical bowel preparation (MBP) was not routinely used. |
|  | Preoperative carbohydrate loading and minimal fasting time | Patients without delayed gastric emptying underwent elective colorectal surgery: 6-h fasting for solids and 2 h for clear fluids including carbohydrates drinks. |
| Postoperative | No nasogastric tubes | No routine use of postoperative nasogastric tube drainage after elective colorectal surgery. |
|  | Postoperative analgesia | Multimodal pain control. |
|  | Supplemental parenteral nutrition | Protein:1.5g/kg ideal body weight, tapered and stopped while enteral nutrition was gradually increased to reach full feeds. |
|  | **Phase I:** 18 h before surgery to 2–4 h before surgery  **Phase II:** POD1 to first flatus or bowel movement  **Phase III:** Two days after phase II  **Phase IV:** from the end of phase III to discharge | **Phase I:** 400 ml carbohydrates  **Phase II:** Liquid diet and oral nutritional  supplements (ONS) as tolerated  **Phase III:** Oral semisolid diet and oral nutritional  **Phase IV:** Supplements (ONS) as tolerated Normal diet |
|  | Early mobilization | Patients were encouraged to mobilize out of bed early after surgery. More details are provided in the Methods section. |
