# Supplementary material for: Insufficient Post-operative Energy Intake Is Associated With Failure of Enhanced Recovery Programs After Laparoscopic Colorectal Cancer Surgery: A Prospective Cohort Study
Source: Front Nutr. 2021 Dec 21;8:768067. doi: 10.3389/fnut.2021.768067 (PMC8724790; doi:10.3389/fnut.2021.768067)
Supplement: Supplementary file 3 [file Table_3.DOCX]

Supplementary Table 3. Details of 6MWD and postoperative activity time.

| POD | 6MWD | Activity time | Both |
| --- | --- | --- | --- |
| POD2 | 2(7.7%) | 3(11.5%) | 2(7.7%) |
| POD3 | 11(42.3%) | 11(42.3%) | 11(42.3%) |
| POD4 | 7(26.9%) | 9(34.6%) | 7(26.9%) |
| POD5 | 2(7.7%) | 5(19.2%) | 2(7.7%) |
| POD6 | 3(11.5%) | 4(15.4%) | 3(11.5%) |
| POD7 | 0 | 1(3.8%) | 0 |
| POD8 | 0 | 0 | 0 |

6MWD: 6-mintue Walking Distance; POD: postoperative day
